# microRNA‐196a‐5p inhibits testicular germ cell tumor progression via NR6A1/E‐cadherin axis

**DOI:** 10.1002/cam4.3498

**Published:** 2020-10-09

**Authors:** Xiaowen Liu, Ziling Fan, Ye Li, Zhilan Li, Zhuan Zhou, Xuehui Yu, Jingyu Wan, Ziqian Min, Lifang Yang, Dan Li

**Affiliations:** ^1^ Institute of Molecular Medicine and Oncology College of Biology Hunan University Changsha P.R. China; ^2^ Cancer Research Institute Xiangya School of Medicine Central South University Changsha P.R. China; ^3^ Department of Pathology Xiangya Hospital Central South University Changsha P.R. China

**Keywords:** E‐cadherin, miR‐196a‐5p, NR6A1, testicular germ cell tumors

## Abstract

Testicular germ cell tumors (TGCTs) are a diverse group of neoplasms that are derived from dysfunctional fetal germ cells and can also present in extragonadal sites. The genetic drivers underlying malignant transformation of TGCTs have not been fully elucidated so far. The aim of the present study is to clarify the functional role and regulatory mechanism of miR‐196a‐5p in TGCTs. We demonstrated that miR‐196a‐5p was downregulated in TGCTs. It can inhibit the proliferation, migration, and invasion of testicular tumor cell lines including NT‐2 and NCCIT through targeting the *NR6A1* gene, which we proved its role in promotion of cell proliferation and repression of cellular junction and aggregation. Mechanistically, NR6A1 inhibited E‐cadherin through binding with DR0 sites in the *CDH1* gene promoter and recruiting methyltransferases Dnmt1. Further, NR6A1 promoted neuronal marker protein MAP2 expression in RA‐induced neurodifferentiation of NT‐2 cells and testicular tumor xenografts. Clinical histopathologically, NR6A1 was positively correlated with MAP2, and negatively correlated with E‐cadherin in TGCTs. These findings revealed that the miR‐196a‐5p represses cell proliferation, migration, invasion, and tumor neurogenesis by inhibition of NR6A1/E‐cadherin signaling axis, which may be a potential target for diagnosis and therapy of TGCTs.

## Graphical abstract

In the present study, we clarified the regulatory pathway of miR‐196a‐5p/NR6A1/E‐cadherin in inhibiting the proliferation, metastasis and neurogenesis of testicular tumor cell in vivo and in vitro, and demonstrated miR‐196a‐5p inhibits testicular tumor progression.

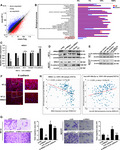

## 1 INTRODUCTION

Testicular germ cell tumors (TGCTs) are the most common type of cancer among young males, with the rising incidence in most parts of the world.^1^, ^2^ In clinical practice of TGCTs, the three common tumor markers including Alpha‐fetoprotein (AFP), b‐human chorionic gonadotrophin (b‐HCG), and lactate dehydrogenase (LDH), are used for diagnosis, risk assessment, and determining patient prognosis.^3^ Nearly 80% of TGCTs patients have been cured with chemotherapy.^4^, ^5^ Unfortunately, 15%–30% of metastatic patients have poor prognosis or even death, suggesting that there might be some unknown abnormal genetic events contributing to TGCTs malignancy.

MicroRNAs (miRNAs/miRs) are endogenous non‐coding RNA molecules composed of 21–25 nucleotides. By post‐transcriptionally modulating corresponding target gene expression, miRNAs can participate in a number of cellular biological processes including cell proliferation, differentiation, apoptosis, metabolism, development and so on.^6^ To date, genome‐wide association studies (GWAS) have identified more than 50 TGCTs susceptibility loci^2^, ^7^, ^8^ and many of the susceptibility loci are in the non‐coding regions of the genome, suggesting that non‐coding RNAs including miRNA may influence the development of TGCTs.^9^, ^10^ The correlation and role of aberrant expression of miRNAs with various types of TGCTs were also investigated. For example, miRNA‐302s, miR‐1297, and miR‐223‐3p may act as oncogenes in TGCTs.^9^, ^10^, ^11^, ^12^ miR‐199a‐5p/3p and miR‐214 in TGCTs can form a self‐regulatory network via PSMD10‐TP53‐DNMT1 and act as tumor‐suppressor.^13^ Thus, miRNAs are expected to result in a breakthrough for diagnosis, prognosis, and therapy of TGCTs.

MiR‐196a‐5p, a conserved miRNA, is derived from its precursor miR‐196a which contains two subtypes: microRNA‐196a‐1 and microRNA‐196a‐2. The former is located between HOXB9 and HOXB13 on chromosome 17, and the latter is located between HOXC10 and HOXC12 on chromosome 12.^14^ In studies of cancers, the HOX family has been reported to be located at genomic instability sites, which are involved in the formation of many tumors,^15^ suggesting that miR‐196a may also be closely related to the pathogenesis of tumors. By functioning as oncogenes in gastrointestinal stromal tumors,^16^ or tumor suppressor genes in malignant melanoma, miR‐196a‐5p has been addressed the association with tumorigenesis. However, its mechanism in TGCTs is still unclear.

Recently, a series of studies demonstrated that tumor cells from human gastric cancer, colorectal cancer, lung cancer, and other tumors have the potential to differentiate into nerve cells, which acts as a crucial part of cancer microenvironment.^17^, ^18^, ^19^ In particular, it was found that interfering with the neural cell generating capability of the tumor cells could significantly inhibit the growth of xenograft tumors in immunodeficient mouse model.^20^ These studies indicated that tumor cells are able to produce one of the most important components, such as functional neurons, in the cancer microenvironment that is required for cancer development and progression.^21^, ^22^, ^23^ In studies of neurodevelopmental diseases, Her LS et al observed that miR‐196a can improve the neuropathology and behavioral phenotype of patients with Huntington's disease.^24^ Thus, we speculate that miR‐196a may play an important role in tumor neurogenesis.

The aim of the current study was to clarify the functional role and regulatory mechanism of miR‐196a‐5p and its targets in TGCTs in vitro and in vivo. Finally, we confirmed that miR‐196a‐5p/NR6A1/E‐cadherin pathway contributes to the inhibition of cell proliferation, migration, invasion, and tumor neurogenesis in TGCTs.

## 2 MATERIALS AND METHODS

### 2.1 Clinical tissue specimens

Clinical formalin‐fixed paraffin‐embedded (FFPE) tissue specimens included 15 TGCTs tissues and 15 adjacent normal testicular tissues, which were collected at the Department of Pathology, Xiangya Hospital of Central South University (Hunan, China). The present study was approved by the Independent Ethical Committee of Xiangya Hospital of Central South University.

### 2.2 Cell culture and all‐trans‐retinoic acid (RA) treatment

The NT‐2 cell line (ATCC CRL‐1973), which was derived from testicular embryonal carcinomas, was monolayer‐cultured routinely: DMEM (Gibco) supplemented with 10% fetal bovine serum (Gibco), 100 units/mL penicillin, and 100 μg/ml streptomycin. The NCCIT cell line (ATCC CRL‐2073), which was derived from testicular embryonal carcinomas as well, was monolayer‐cultured routinely: 1640 (Gibco) supplemented with 10% fetal bovine serum (Gibco), 100 units/ml penicillin, and 100 μg/ml streptomycin. RA (Sigma‐Aldrich) was used as an inducer for cell differentiation. For the establishment of RA‐induced neurodifferentiation model in vitro, NT‐2 or NCCIT cells were seeded and grown to an 80% confluent monolayer. Two days later, cells were digested into single‐cell suspensions and cultured in 1% agarose dishes to form embryoid bodies. The embryoid bodies were digested with trypsin, and the cells were continued in adherent culture with 10 μM RA for 21 days until showing significant changes in cell morphology.

### 2.3 Lentivirus preparation

Lentivirus vector pLv[Exp]‐EGFP:T2A:Puro‐EF1A>NR6A1 containing human NR6A1‐coding sequence, was purchased from Guangzhou Trauer Biotechnology Co.LTD. Three plasmids, the Gag‐pol, Rev, and VSV‐G plasmids (Guangzhou Trauer Biotechnology) were used for packaging lentivirus vectors. Cells were collected after transient transfection of lentivirus vector into HEK293T cells for 48 h or 72 h using TurboFect^TM^ in vitro Transfection Reagent (Fermentas), respectively. Then supernatants were concentrated and filtered at 4℃. The viral titer was determined to be 10^8^ TU/ml by counting EGFP‐positive cells with flow cytometry.

### 2.4 Transient transfection

The transient transfection was performed with TurboFect^TM^ in vitro Transfection Reagent (Fermentas). The final concentration of 50 nmol/L for miRNA mimics or inhibitors, and 100 nmol/L for si‐RNA were used. The sequences of the miR‐196a‐5p mimics, inhibitors, and the si‐CDH1 or si‐NR6A1 were all synthesized from Genepharma Company in Shanghai, China.

### 2.5 Cell viability assay

3‐(4, 5)‐dimethylthiahiazo‐3, 5‐diphenytetrazoliumromide (MTT, Sigma) was added to 96 wells and incubated (at a concentration of 500 mg/L) for 4 h after cells treatment for different days. The 150 μl/well of dimethyl sulfoxide was used for dissolution of formazan. Opacity density (OD) was measured at 570 nm using a microplate reader (Thermo).

### 2.6 Wound healing assay

Wound healing assay was performed to examine the cell migration ability. The 90% confluent cells were subsequently scraped with a sterile 100 µl pipette tip and the cell debris was washed with D‐hanks solution. Images were captured under bright field light microscopy with a Nikon Eclipse E600 microscope at 0 h and 48 h after scraping.

### 2.7 Transwell assay

Serum‐free medium was used to dilute individual suspended cells, and cells were added into upper chamber of the 24‐well insert with 8 mm pore size. Then the cells were incubated for 24 h before stained with 20% crystal violet. After re‐movement of metastatic cells by cotton swab from the upper compartment, stained cells were counted under a microscope in five randomly chosen fields and the average number was calculated. For invasion analysis, the upper chamber was pre‐coated with 80 μl of Matrigel solution (BD, USA) and the lower chamber was added with 800 μl of 10% FBS medium before cells were added into upper chamber.

### 2.8 Total RNA extraction and quantitative polymerase chain reaction (qPCR)

The total RNA from clinical FFPE tissue specimens were isolated by RecoverAll^TM^ Total Nucleic Acid Isolation kit (Ambion, Thermo Fisher Scientific), according to the manufacturer's protocol. Total RNA from cultured cells was extracted by RNAiso (Takara) and treated by DNase I (Fermentas), then applied for miRNA reverse transcription by one step PrimeScript miRNA cDNA Synthesis kit (Takara), or for mRNA reverse transcription by PrimeScript RT reagent kit with gDNA Eraser (Takara). qPCR was performed using an SYBR Premix Ex Taq II kit and the MX3000 instrument (Stratagene). The following is thermocycling condition: Initial denaturation, 95°C for 10 min; 40 cycles of 95°C for 10 s, 60°C for 30 s and 72°C for 32 s. U6 was used as an internal control for miRNA analysis, and *β*‐*actin* was used as an internal control for mRNA analysis. 2^−ΔΔCt^ was the ratio of gene expression of experimental group and the control group.

Primer sequences were as follows: *β*‐*actin* F: CCAACCGCGAGAAGATGA, R: CCAGAGGCGTACAGGGATA; *NR6A1* F: CCCTCCGATGAAGAACTACACAGAT, R: GCATACTCCTCGTTGCTGACCT; *MAP2* F: CCTGTGTTAAGCGGAAAACC, R: AGAGACTTTGTCCTTTGCCTGT; *OCT4* F: GGAGCCCTGCACCGTCA, R: ATGGTCGTTTGGCTGAAT; *CDH1* F: GCCCTGCCAATCCCGATGAAA, R: GGGGTCAGTATCAGCCGCT; *CDH2* F: TCAGGCGTCTGTAGAGGCTT, R: ATGCACATCCTTCGATAAGACTG; miR‐196a‐5p: TAGGTAGTTTCATGTTGTTGGG. miRNA reverse primer and U6 primers were provided by the one step PrimeScript miRNA cDNA Synthesis kit (Takara).

### 2.9 Western blotting

Proteins were extracted using ice‐cold RIPA (Beyotime Biotech) supplemented with protease inhibitors (Selleck.cn) and PMSF (Biotool Biotech) at 4°C for 30 min. Protein concentration was detected by bicinchoninic acid (BCA) method (Dingguo Biotech). Proteins (50 ug) were electrophoresed in 10% SDS gel electrophoresis, transferred onto PVDF membrane (Millipore, USA), and blocked for 1 h with 5% nonfat milk at room temperature. The membranes were, respectively, incubated with antibodies against NR6A1 (1:1000, CST, #5417), E‐cadherin (1:1000, CST, #3195), N‐cadherin (1:1000, CST, #4061), OCT4 (1:500, Beyotime Biotech, AF2506), MAP2 (1:1000, Sangon Biotech, AF2215) at 4℃ overnight, then with secondary antibody at room temperature for 1 h. The blots were detected using enhanced chemiluminescence (Bio‐Rad). β‐actin (1:5000, Bioworld Technology, AP0714) served as the internal control.

### 2.10 Dual‐luciferase reporter assay

Potential miR‐196a‐5p binding sites of NR6A1 3′‐UTR were predicted by Targetscan (http://www.targetscan.org/). The fragment of NR6A1‐3′‐UTR‐wild‐type (NR6A1‐3′‐UTR‐wt) with miR‐196a‐5p binding sites and its mutant (NR6A1‐3′‐UTR‐mut) were digested with restriction enzyme Xho I and Not I, then cloned into pmiR‐RB‐REPORT plasmids (Guangzhou RiboBio), respectively. Then the recombinant plasmids and miR‐196a‐5p mimics/NC mimics were co‐transfected into HEK293T cells using TurboFect^TM^. Relative Renilla activities were measured 48 h after transfection and firefly luciferase activity was normalized by luciferase activity.

The different truncations of *CDH1* promoter containing three DR0 sites were PCR amplified by high fidelity enzyme (Transgen biotech) using human genomic DNA as template, then, respectively, cloned into pGL3 basic reporter plasmids (Promega) by double digestion of Kpn I and Hind III. All recombinant plasmids were sequenced and have no mutation. Recombinant naming was based on the positions of the promoter fragments. The recombinant plasmids contained −1938 to −1927 (pGL3 *CDH1*‐a), −912 to −917(pGL3 *CDH1*‐b), −167 to −178 (pGL3 *CDH1*‐c). All the recombinant plasmids were mixed with Renilla vectors, then co‐transfected into HEK293T cells with NR6A1. The relative fluorescence value is calculated by ratio of Luc/Ren.

### 2.11 Chromatin Immunoprecipitation (ChIP)

Briefly, NT‐2 cells were cross‐linked with 1% formaldehyde for 10 min at 37°C and terminated by 0.125 mol/L glycine at room temperature, then lysed by SDS lysis. Samples were sonicated on ice in 25% power, 5 s pulse, 9 s interval, 14 times in total using Bioruptor Pico (Diagenode s.a., Seraing, Belgium) to obtain ~1000 bp fragments. After centrifugation, the supernatant was diluted with CHIP dilution buffer and pre‐cleared with 80 µL protein A/G magnetic beads at 4°C for 4 h. The lysates were incubated with 5 µg anti‐Flag (Sigma, F2555), or control anti‐IgG (Santa Cruz, sc‐2025) overnight at 4°C. The immunocomplexes were then collected with protein magnetic beads. The magnetic beads were, respectively, washed three times in different buffers. The immunocomplexes were eluted and de‐crosslinked at 65°C overnight. After RNase (Thermo Fisher Scientific, Inc.) digestion at 37°C and proteinase K (Thermo Fisher Scientific, Inc.) digestion at 45°C, the immunoprecipitated DNA was extracted and amplified by qPCR as aforementioned. Primer sequences for CHIP‐qPCR were as follows: *CDH1* DR0 (a) F: CTGAGGCAGGTGGATCATCT, R: CCACCACGACTGGCTAATTT; *CDH1* DR0 (b) F: CAGTGGCTCACGCCTGTAAT, R: CATGGTGAAACCCCGTCTGT; *CDH1* DR0 (c) F: ACCCAGTGGAATCAGAACCG, R: TAGAGGGTCACCGCGTCTAT. Besides, the primers: 5′‐ACCTCCCTCTCCTCCACCCAT‐3′(Forward), and 5′‐GAAGGGACTACTCAACCCCTCTCT‐3′(Reverse) were used for *OCT4* promoter amplification as positive control.

### 2.12 Co‐Immunoprecipitation (Co‐IP)

In brief, total proteins were extracted from NT‐2 cells with Western and IP lysis buffer (Beyotime Biotech) and precleared with 30 μl protein A/G magnetic beads (Selleck) at 4°C. Meantime, 50 μl magnetic beads, mixed with 3 μg anti‐Flag antibodies (Sigma, F2555) or control IgG antibodies (Santa Cruz, sc‐2025), were pre‐incubated for 4 h at 4°C before immunoprecipitated with total proteins at 4°C overnight. Then beads were washed with Washing buffer (Beyotime Biotech, China) three times for 15 min, and resuspended in 50 μl lysis buffer with 1×SDS loading buffer, and boiled for 10 min.

### 2.13 RNA‐Sequencing and Gene Ontology (GO) enrichment analysis

High throughput RNA sequencing was performed by Shanghai Origin‐gene Biological Company. Briefly, HiSeq platform was used to sequence all mRNAs transcribed from NT‐2 cells or stable transfection NT‐2‐NR6A1 cells, respectively. And the complementary DNA (cDNA) library for sequencing was conducted by Illumina Truseq^TM^RNA sample prep Kit. GO Enrichment Analysis^25^ provides all of the GO terms that are significantly enriched in differentially expressed genes (DEGs) and filters the DEGs that correspond to biological functions (http://www.geneontology.org/). The calculated P‐value is subjected to the Bonferroni correction, using the corrected *p*‐value ≤0.05 as a threshold.

### 2.14 Immunofluorescence analysis

Cells were fixed with 4% paraformaldehyde (Sigma) for 15 min and permeated with 0.2% Triton X‐100 for 5 min. After washed with PBS buffer three times for 5 min, cells were blocked with fetal bovine serum for 30 min, then incubated with the primary antibody at 4°C overnight [E‐cadherin (1:100, CST, #3195); MAP2 (1:40, Sangon Biotech, AF2215)]. Cells were washed with PBS buffer three times for 5 min and incubated with fluorescence secondary antibody (1:1000, sigma) for 30 min. After washing with PBS buffer, the cells were stained with DAPI (Thermo Fisher Scientific) for 10 min and washed with PBS buffer three times for 5 min before photographed with Inverted Fluorescence Microscope.

### 2.15 Immunohistochemistry (IHC) and correlation analysis

IHC was performed according to standard procedures. The paraffin sections of xenograft were randomly selected to detect all biomarkers. For clinical FFPE tissue specimens, the tissue slices were randomly selected from the same one to detect all biomarkers. The primary antibodies anti‐NR6A1 (1:50, Proteintech, China, 12712‐1‐AP), anti‐MAP2(1:8000, Abcam, ab1838830), anti‐PCNA (1:100, WanleiBio, China, WL03213,), anti‐E‐cadherin (1: 200, CST, #3195), and DAB (ZSGB‐Bio, China) were used for staining. The results were obtained by digital slice scanner (3DHISTECH, Hungary). The staining score of IHC was estimated as negative (0), weak (1), moderate (2), and strong (3). The extent of staining, defined as the percentage of positive stained cells, was scored as 1 (≤10%), 2 (11%−50%), 3 (51%−80%), and 4 (> 80%). The total immune reactive score (IRS) was obtained by multiplying the score of intensity and that of extent, ranking from negative (−) to >6 (+++).^26^

### 2.16 In vivo xenograft experiment

The animal studies were approved by the Experimental Animal Ethics Committee of College of Biology, Hunan University. Six‐week‐old female nude mice (BALB/C) were injected subcutaneously with stable transfected 4 × 10^7^ NT‐2 cells infected by NR6A1 or empty vector lentivirus, and five mice in each group. Tumor formation was investigated every 3 days, the tumor volume was calculated using the formula *V* = *a* × *b* ^2^/2, where “*a*” is the long axis and “*b*” is the short axis. After 19 days of subcutaneous injection, mice were killed and the tumor was embedded into paraffin tissue sections for IHC detection.

### 2.17 Statistics

All quantitative data are presented as mean ±standard deviation. In tissue samples, clinicopathological characteristics and NR6A1/E‐cadherin/MAP2 expression was analyzed using the *χ* ^2^ test. The relationship analysis based on IHC score between NR6A1/E‐cadherin/MAP2 was performed by the Pearson method. The means of data between two groups were compared by two‐sided Student's *t* tests. One‐way ANOVA was used for comparisons of multiple independent groups. All assays were repeated independently three times, and representative images are shown. Values of *p* < 0.05 were considered significant, *p* < 0.01 were considered prominent significant.

## 3 RESULTS

### 3.1 MiR‐196a‐5p inhibits the proliferation, migration, and invasion of NT‐2 and NCCIT cells

**FIGURE 1:**
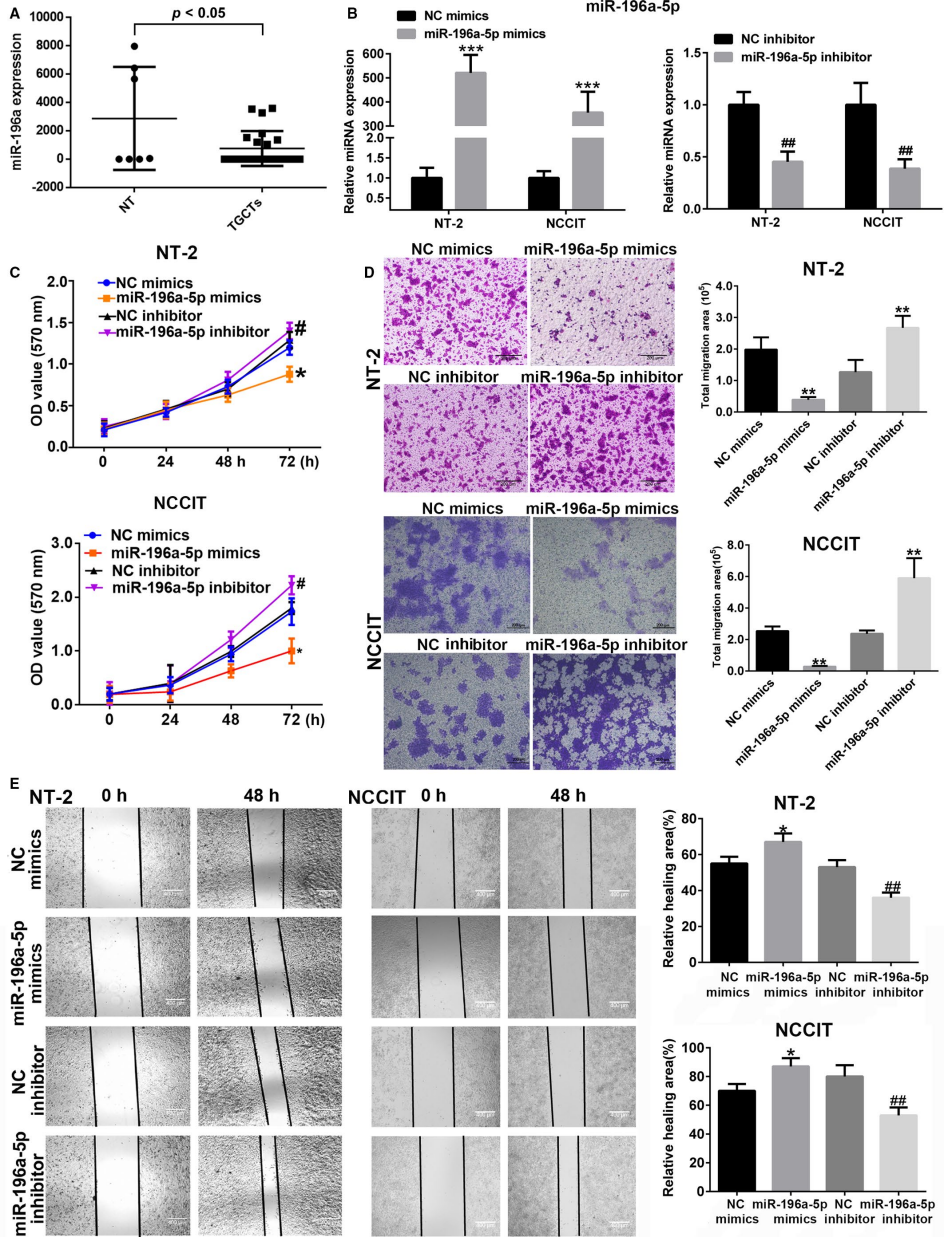
miR‐196a‐5p inhibited the proliferation and migration of NT‐2 and NCCIT cells. A, The expression alteration of miR‐196a‐5p in both normal testes and testicular tumors by GEO database analysis. NT: normal testes; TGCTs: testicular germ cell tumors. B, Measurement of miR‐196a‐5p mimics or inhibitor transfection efficiency by RT‐qPCR in NT‐2 and NCCIT cells. C, The inhibition of miR‐196a‐5p on the proliferation of NT‐2 and NCCIT cells by MTT analysis. D, The inhibition of miR‐196a‐5p on the migration of NT‐2 and NCCIT cell by transwell assay analysis. E, The inhibition of miR‐196a‐5p on the migration of NT‐2 and NCCIT cell by wound healing assay analysis. * or # *p* < 0.05, ** or ## *p* < 0.01, ***p < 0.001. * is compared with NC mimics group and # is compared with NC inhibitor group. All data are shown as the means ±SDs of three independent experiments

To determine whether the miR‐196a‐5p involved in TGCTs, we analyzed the expression alteration of miR‐196a‐5p between normal testes and TGCTs using GEO database (GEO accession: GSE59267, GSE31824). The data showed that the expression of miR‐196a‐5p is down‐regulation in TGCTs (Figure 1A). By RT‐qPCR, we confirmed that the transfection efficiency of miR‐196a‐5p mimics or inhibitor was effective in testicular embryonal carcinoma cells NT‐2 and NCCIT (Figure 1B). Then, MTT assay revealed that compared with the control NC group, miR‐196a‐5p mimics significantly inhibited the proliferation of NT‐2 and NCCIT cells after 72 h of transfection, whereas the miR‐196a‐5p inhibitor promoted cell proliferation (Figure 1C). In vitro transwell (Figure 1D) and wound healing (Figure 1E) assays showed that miR‐196a‐5p mimics inhibited the migration of NT‐2 and NCCIT cells, whereas the results were reversed after treatment with the miR‐196a‐5p inhibitor. And we also investigated the inhibition of miR‐196a‐5p on the invasion of NT‐2 cells (Figure S1). These results indicated that miR‐196a‐5p inhibits the proliferation, migration, and invasion of testicular embryonal carcinoma cells.

### 3.2 MiR‐196a‐5p targets NR6A1 for inhibition

**FIGURE 2:**
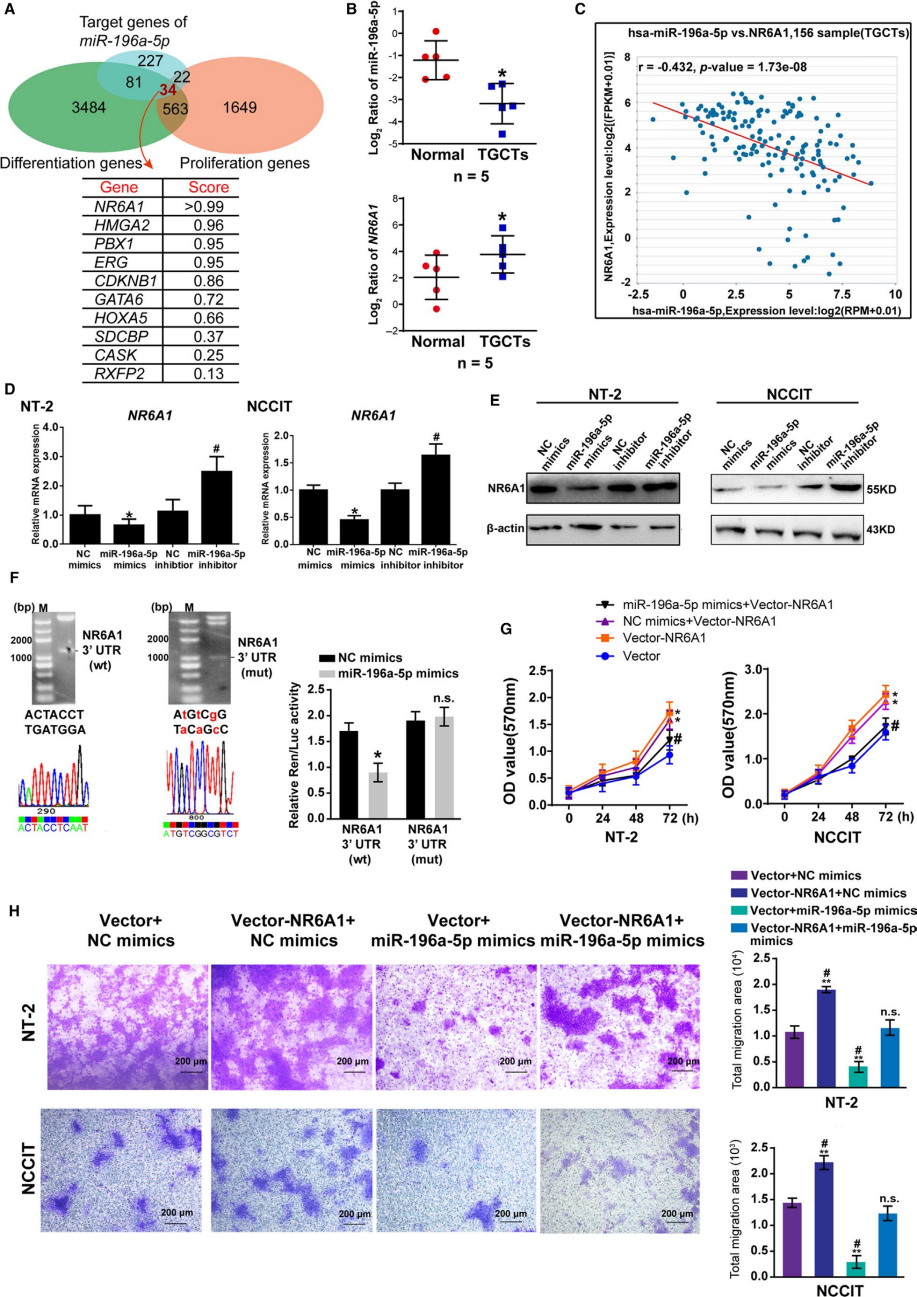
miR‐196a‐5p targeted NR6A1 for inhibition. A, The screening of target genes for miR‐196a‐5p. Venn diagram showing overlapping genes among 364 target genes predicted by TargetScan and 4162 proliferation genes and 2268 differentiation genes from the GO database. Among these genes, 34 target genes were selected. The nuclear receptor NR6A1 was ranked first among these 34 target genes, with a score of greater than 0.99. B, The reciprocal expression of miR‐196a‐5p and NR6A1 in clinical normal testes tissues and TGCTs by RT‐qPCR, *p < 0.05. C, The correlation analysis between miR‐196a‐5p and NR6A1 in TGCTs. *r* = −0.432, *p* = 1.73e‐08. The correlation is deemed significant and positive when *p* < 0.05. D, RT‐qPCR verification of the inhibitory effect of miR‐196a‐5p on *NR6A1* mRNA expression in both NT‐2 and NCCIT cells. * represented *p* < 0.05 compared with NC mimics group and # represented *p* < 0.05 compared with NC inhibitor group. E, Western blot analysis of the inhibitory effect of miR‐196a‐5p on NR6A1 protein expression in both NT‐2 and NCCIT cells. F, Analysis of the binding between miR‐196a‐5p and NR6A1 by the luciferase reporter assay. Left: Double restriction enzyme digestion and sequence analysis of pmiR‐RB‐REPORT NR6A1‐3'UTRwt and its mutant (red indicates mutated bases); Right: Recombinant reporter vector and miR‐196a‐5p mimics were cotransfected into HEK293T cells for assessment of renilla and luciferase activity, and the final fluorescence value was obtained as Ren/Luc, * represented *p* < 0.05 compared with NC mimics group. n.s. represented no significant changes. G, Cooverexpression of NR6A1 and miR‐196a‐5p mimics rescued the proliferation of NT‐2 and NCCIT cells, as evidenced by the MTT assay. *represented *p* < 0.05 compared with Vector group, and # represented *p* <0.05 compared with NC mimics + Vector‐NR6A1 group. H, Cooverexpression of NR6A1 and miR‐196a‐5p mimics rescued the migration ability of NT‐2 and NCCIT cells, as evidenced by the transwell assay. ** represented *p* < 0.01 compared with Vector +NC mimics group, and # represented *p* < 0.05 compared with Vector‐NR6A1 + miR‐196a‐5p mimics group; n.s. represented no significant changes compared with Vector + NC mimics group. All data are shown as the means ± SDs of three independent experiments [Correction added on 29 October 2020, after first online publication: In Figure 2B, "Log2 Ratio of miR‐196a‐5p" has been corrected from "Log2 Ratio of NR6A1" and In legend, line 14: "and # represented *p* <0.05 compared with NC mmimics + Vector‐NR6A1 group" has been corrected from "and # represented *p* <0.05 compared with NC mimics + Vector‐NR6A1 group" in this version]

Generally, miRNA exerts an inhibitory effect by binding to the 3'UTR sequence of the target gene mRNA.^27^ We downloaded all predicted target genes (364) of miR‐196a‐5p from TargetScan and analyzed their function via GO databases, and the results showed that most miR‐196a‐5p target genes are involved in nuclear processes, metabolism, biological regulation, proliferation, development, and biological adhesion (Figure S2A), indicating that miR‐196a‐5p plays multiple roles through targeting different genes. To investigate the relationship between miR‐196a‐5p and cell proliferation and differentiation, we mapped the 364 predicted target genes to 4162 proliferation genes and 2268 differentiation genes from the GO database and screened 34 target genes related to cell proliferation and differentiation. Among these genes, nuclear receptor NR6A1 was ranked first, with a score of greater than 0.99 (Figure 2A). TargetScan analysis predicted that the 3'UTR of *NR6A1* mRNA contained four sites that were recognized by miR‐196a‐5p seed sequences (Figure S2B, only the first one is shown), and the hybridization free energy was much lower than its free energy of binding to self mRNA (Figure S2C). The analysis of NCBI GEO datasets for mice on different days of development showed a negative correlation of the spatiotemporal expression profile between miR‐196a‐5p and *NR6A1* (Figure S2D). The above bioinformatics and database analysis showed that *NR6A1* was highly likely to be a target gene of miR‐196a‐5p.

Subsequently, RT‐qPCR data showed the reciprocal expression between miR‐196a‐5p and *NR6A1* in both normal testes and TGCTs derived from clinical patients (Figure 2B). The expression correlation analysis of miR‐196a‐5p and NR6A1 based on starbase (http://starbase.sysu.edu.cn/) was significant (Figure 2C, *r* = −0.432, *p* = 1.73e‐08). We also verified that miR‐196a‐5p can inhibit the expression of NR6A1 at both the mRNA (Figure 2D) and protein (Figure 2E) levels in both NT‐2 and NCCIT cells. Then, the *NR6A1* 3'UTR sequence containing the binding sites for miR‐196a‐5p and its mutant (Figure 2F, left: the red color indicates the mutated bases) were amplified and inserted into the luciferase reporter gene vector pmiR‐RB‐REPORT. The constructed recombinant reporter and miR‐196a‐5p mimics or NC mimics were cotransfected into HEK293T cells for assessment of luciferase activity. As shown in Figure 2F (Right), miR‐196a‐5p mimics significantly inhibited the luciferase activity in the *NR6A1*‐3’UTR‐wt group, but no influence was observed in the *NR6A1*‐3'UTR‐mut group, implying that miR‐196a‐5p directly recognized and inhibited NR6A1. Further, we constructed the pLv[Exp]‐EGFP: T2A: Puro‐EF1A>NR6A1 lentiviral vector, and NR6A1 overexpression were successful and efficient in HEK293T, NT‐2 and NCCIT cells (Figure S3A,B). The functional rescue assay in NT‐2 and NCCIT cells confirmed that co‐overexpression of miR‐196a‐5p and NR6A1 partially restored the inhibitive effect of miR‐196a‐5p on cell proliferation (Figure 2G) and migration (Figure 2H). Co‐overexpression of miR‐196a‐5p and NR6A1 also partially restored the inhibitive effect of miR‐196a‐5p on cell invasion in NT‐2 cells (Figure S3C). These results indicated that NR6A1 is a functional target gene of miR‐196a‐5p in TGCTs.

### 3.3 NR6A1 inhibits cell junction by targeting E‐cadherin

**FIGURE 3:**
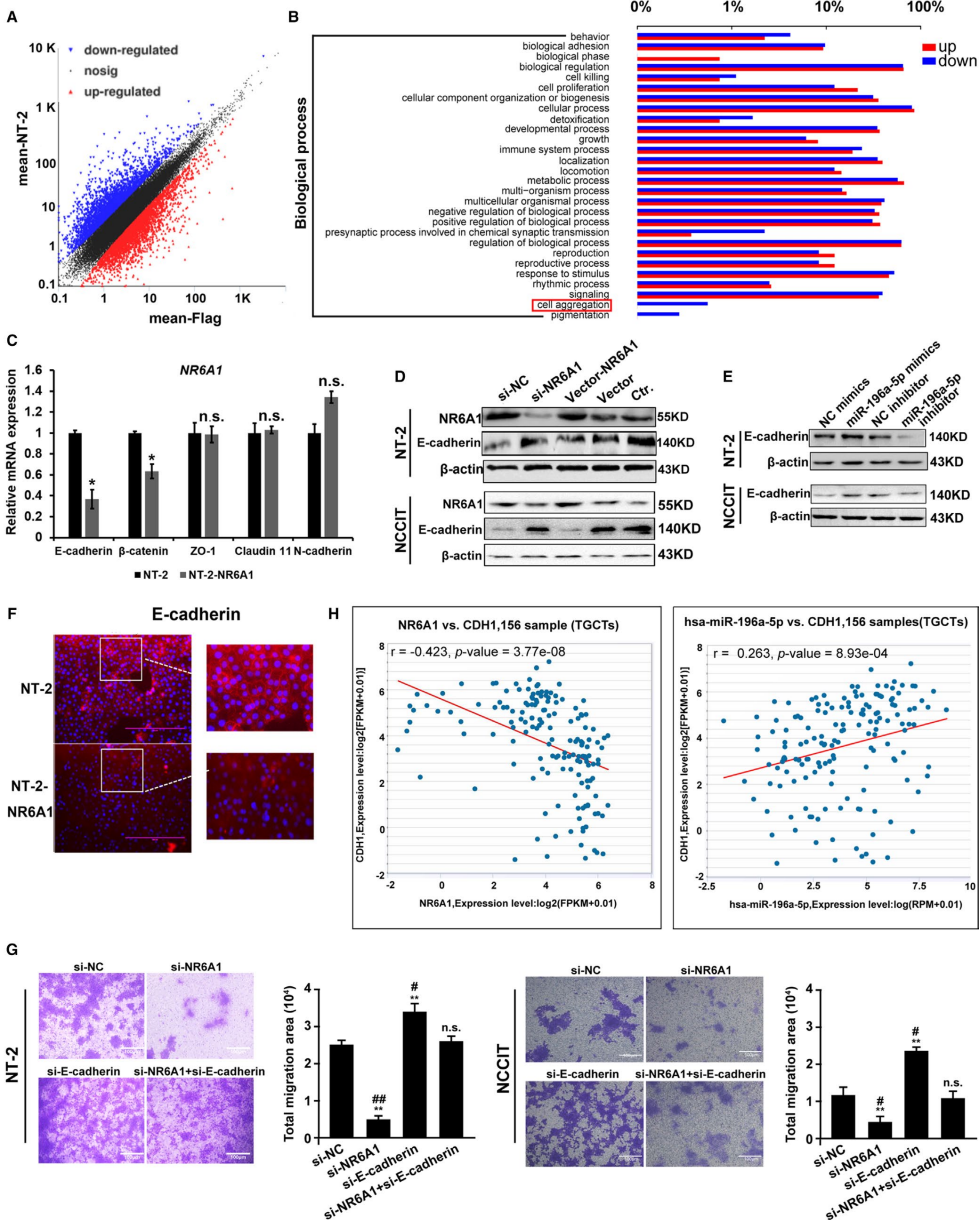
NR6A1 repressed cell junction by targeting E‐cadherin. A, The log‐log scatter‐plot analysis of RNA‐sequencing of NT‐2 cells transfected with NR6A1. B, Biological process analysis of RNA‐sequencing results of NT‐2 cells transfected with NR6A1 by GO enrichment analysis. The red rectangle indicates that NR6A1 down‐regulated the cell aggregation. C, The RT‐qPCR analysis of cell junction genes in NT‐2 cells with NR6A1 overexpression, *p < 0.05, n.s. represented no significant changes. D, Western blot analysis of E‐cadherin alteration after NR6A1 knockdown or overexpression in both NT‐2 and NCCIT cells. E, Western blot analysis of E‐cadherin alteration after miR‐196a‐5p knockdown or overexpression in both NT‐2 and NCCIT cells. F, Immunofluorescence analysis of E‐cadherin alteration in NT‐2 cells with NR6A1 overexpression. G, The effect of co‐interference with NR6A1 and E‐cadherin on the migration ability of NT‐2 and NCCIT cells by a transwell assay analysis. ** represented *p* < 0.01 compared with si‐NC group, and # represented *p* < 0.05 compared with si‐NR6A1 + si‐E‐cadherin group; n.s. represented no significant changes compared with si‐NC group. (H) The correlation analysis between *NR6A1* and *CDH1* (R = −0.423; *p* = 3.77e‐08), miR‐196a‐5p and *CDH1* (R = 0.263; *p* = 8.93e‐04.) in TGCTs, respectively. The correlation is deemed significant and positive when *p* < 0.05. All data are shown as the means ±SDs of three independent experiments [Correction added on 29 October 2020, after first online publication: In Figure 3E,"The molecular weight of *β*‐actin in NT‐2 cell: 140 KD has been corrected from 43 KD"; In Figure 3H, figure on the right: "r = ‐0.263 has been corrected from r = 0.263" and In Legend of Figure 3, line 10: "miR‐196a‐5p and *CDH1* (R = ‐0.263; *p* = 8.93e‐04.) in TGCTs" has been corrected from "miR‐196a‐5p and *CDH1* (R = 0.263;*p* = 8.93e‐04.) in TGCTs" in this version]

In order to clarify the role of NR6A1 in TGCTs, the global gene expression levels induced by NR6A1 in NT‐2 cells were analyzed by RNA‐sequencing. The differentially expressed genes with fold changes in ≥2 or ≤0.5 (*p* ≤ 0.05) were presented by scatter‐plot result (Figure 3A). The data showed that the expression of 858 out of 15008 genes was altered after NR6A1 was overexpressed. Of these, 362 genes were upregulated and 496 genes were downregulated specifically (data not shown). The biological process by GO analysis (Figure 3B) implied that NR6A1 has the greatest impact on cell aggregation in a negative way (The red rectangle indicated). Subsequently, the expression of several typical cell junctions and adhesion genes was measured by RT‐qPCR and results showed that the *CDH1* (coding gene for E‐cadherin) mRNA levels gradually decreased in NR6A1 lentivirus‐infected NT‐2 cells (Figure 3C), which is consistent with the data of RNA‐sequencing. At protein level, overexpression of NR6A1 significantly inhibited the expression of E‐cadherin in both NT‐2 and NCCIT cells as well (Figure 3D). In addition, upregulation of E‐cadherin was observed in the miR‐196a‐5p‐treated group (Figure 3E). Immunofluorescence analysis also showed that E‐cadherin expression and the cell junction were significantly decreased in NR6A1 lentivirus‐infected NT‐2 cells (Figure 3F). Transwell assay data (Figure 3G) showed that interference with NR6A1 inhibited the migration behavior of NT‐2 and NCCIT cells, while the impact of si‐E‐cadherin on cell migration was the inverse, consistent with the function of E‐cadherin as an adhesion protein. Additionally, interference with both NR6A1 and E‐cadherin restored the cell migration ability induced by si‐E‐cadherin alone. The inhibition of si‐NR6A1 on invasion behavior of NT‐2 and the functional rescue of si‐E‐cadherin were also investigated (Figure S4). Finally, the expression correlation analysis of *NR6A1* and *CDH1* (*r* = −0.423, *p* = 3.77e‐08), miR‐196a‐5p and *CDH1* (r = 0.263, *p* = 8.93e‐04), based on starbase (http://starbase.sysu.edu.cn/) was statistically significant (Figure 3H), implying their regulation relationship of miR‐196a‐5p, NR6A1 and E‐cadherin [Correction added on 29 October 2020, after first online publication: In section 3.3, text: "(r = −0.263, *p* = 8.93e‐04), based on starbase (http://starbase.sysu.edu.cn/) has been corrected from (r = 0.263, *p* = 8.93e‐04), based on starbase (http://starbase.sysu.edu.cn/)" in this version].

### 3.4 NR6A1 represses E‐cadherin via binding with DR0 sites and recruiting Dnmt1

**FIGURE 4:**
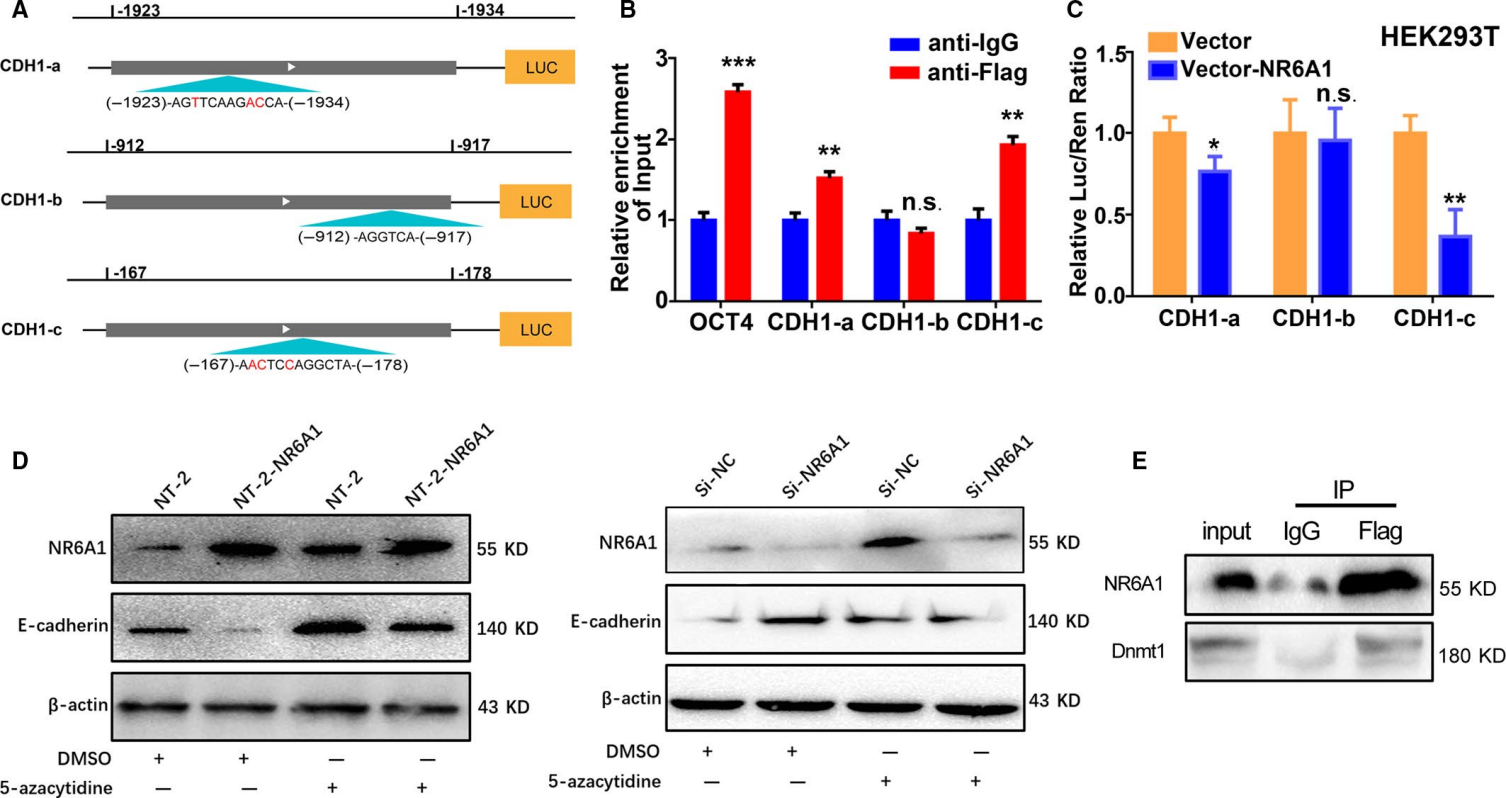
NR6A1 targeted E‐cadherin via binding with DR0 sites and recruiting Dnmt1. A, Schematic diagram of the *CDH1* promoter region. The DR0 sites at −1923 to −1934 (fragment CDH1‐a) and −167 to −178 (fragment CDH1‐c), contain three base mutations (red indicates the mutated base sites); the DR0 site −912 to −917 (fragment CDH1‐b) contains an unmutated half‐site (AGGTCA). B, CHIP‐qPCR analysis of NR6A1 enrichment at the *CDH1* and *OCT4* promoter DR0 sites. Anti‐Flag antibody was used to immunoprecipitate NR6A1 protein from NT‐2 cells, and IgG antibody was used as the control. **p < 0.01, ***p < 0.001, compared with anti‐IgG group. n.s. represented no significant changes. C, Analysis of target recognition of NR6A1 and *CDH1* promoter by the luciferase reporter system. Three *CDH1* promoter fragments containing DR0 sites were inserted into the pGL3‐Basic reporter vector and then cotransfected with NR6A1 into HEK293T cells. The relative luciferase activity (Luc/Ren) was then measured and calculated. The error bars indicate the SDs of three independent experiments. *p < 0.05, **p < 0.01. * is compared with vector group. n.s. represented no significant changes. D, Western blot analysis of E‐cadherin in NT‐2 cells with NR6A1 overexpression or knockdown after DNA methyltransferase inhibitor 5‐azacytidine treatment. E, Co‐immunoprecipitation analysis of the interaction between NR6A1 and Dnmt1. Anti‐Flag antibody was used to immunoprecipitate NR6A1 in NR6A1‐overexpressing NT‐2 cells, and NR6A1 and Dnmt1 expression was assessed by western blotting. All data are shown as the means ±SDs of three independent experiments

NR6A1 plays a role as a transcriptional repressor by binding to an evolutionarily conserved DR0 element in the promoter of its target gene—AGGTCAAGGTCA (or AGGCTAAGGCTA)—or to an extended half DR0 site (AGGTCA).^28^, ^29^ To confirm whether NR6A1 directly targeted *CDH1* via binding to DR0 sites, we analyzed the *CDH1* gene promoter. Three DR0 sites were predicted (Figure 4A). Among these DR0 sites, the −1938 to −1927 (fragment *CDH1*‐a) and −912 to −917 (fragment *CDH1*‐b) fragments contained three mutated bases (the red markers indicate the mutated base sites), and −167 to −178 (fragment *CDH1*‐c) contained an unmutated half‐site (AGGTCA). The CHIP‐qPCR assay was performed to determine whether NR6A1 is enriched on the promoter of *CDH1*, and the results showed that compared with the anti‐IgG group, the anti‐Flag group exhibited enrichment in the *CDH1* promoter (fragment a, c) and *OCT4* promoter (as a positive control), while *CDH1*‐b was not enriched (Figure 4B). This finding indicated that NR6A1 can directly bind to the DR0 site in the *CDH1* gene promoter. Further, the *CDH1* gene promoter sequences containing the abovementioned three DR0 sites were inserted into the pGL3‐Basic reporter vector and transfected into HEK293T cells for assessment of luciferase (Luc/Ren) activity. As shown in Figure 4C, the luciferase activity of the *CDH1* promoter containing both the a and c fragments was obviously repressed by NR6A1, implying the inhibitory effect of NR6A1 on *CDH1* gene. It has been reported that NR6A1 may play an inhibitory role by interacting with Dnmt3L.^30^ After being treated with 5‐azacytidine, a DNA methyltransferase inhibitor, we found that the expression of E‐cadherin was enhanced, and the presence of NR6A1 could weaken the enhancement, suggesting that the E‐cadherin might also be inhibited by NR6A1 in a way of DNA methylation (Figure 4D). Subsequently, we confirmed the interaction between NR6A1 and Dnmt1 by Co‐IP experiment, which indicated that NR6A1 inhibited the expression of E‐cadherin by recruiting Dnmt1 (Figure 4E). The above results suggest that NR6A1 inhibits *CDH1* by binding with DR0 sites and recruiting Dnmt1.

### 3.5 NR6A1 promotes neural cell‐like characteristics of testicular tumor in vivo and in vitro

**FIGURE 5:**
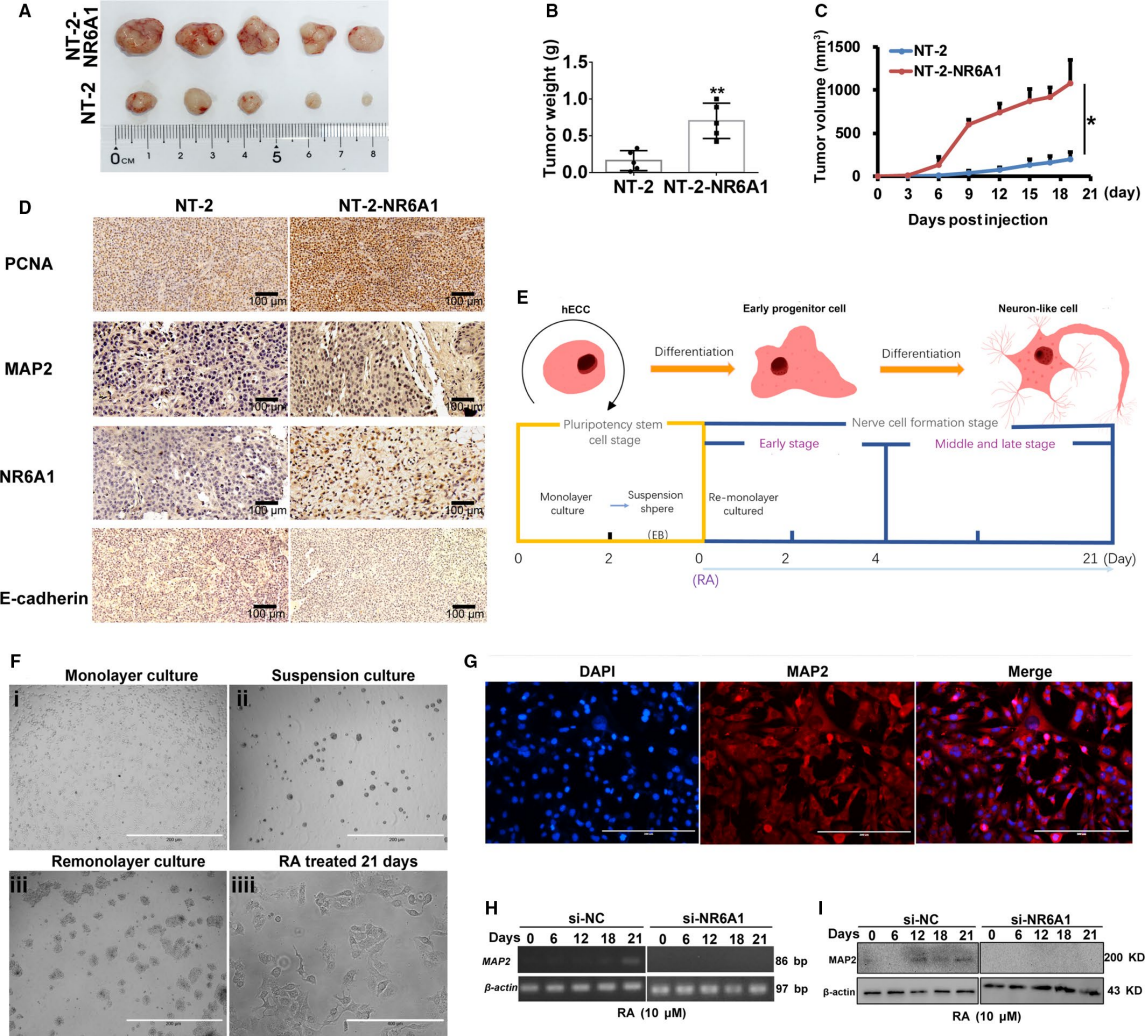
NR6A1 promoted neural cell‐like characteristics of testicular tumor in vivo and in vitro. A, At the experimental end point, tumor xenografts were dissected and photographed. B, Tumor weights were measured at the experimental end point, **p < 0.01. C, Tumor volume was measured every 3 days after injection of tumor cells, *p < 0.05. D, Immunohistochemistry staining of NR6A1, PCNA, E‐cadherin, and MAP2 protein in NT‐2 testicular tumor xenografts. E, Diagram for establishment of RA‐induced NT‐2 cell neurodifferentiation model in vitro. F, The morphological changes in NT‐2 cells under different RA induction days. G, Immunofluorescence analysis of the expression and localization of MAP2 protein in NT‐2 cells with RA induction of 21 days. H, RT‐PCR analysis of MAP2 in long‐term RA‐induced NT‐2 cells with NR6A1 interference. I, Western blot analysis of MAP2 in long‐term RA‐induced NT‐2 cells with NR6A1 interference. All data are shown as the means ±SDs of three independent experiments

The gain of neural cell‐like characteristics of cancer cells is reported to be closely related to cancer progression.^31^ In order to determine the involvement of NR6A1 in neurogenesis of the tumors that were generated from human NT‐2 testicular tumor cells in vivo, we transplanted the NT‐2 cells that were transfected with either NR6A1 or empty vector into nude mice via subcutaneous injections to produce human testicular tumor xenografts. The xenograft tumors derived from NR6A1‐overexpressing NT‐2 cells had significantly higher growth rates than controls (Figure 5A‐C). The IHC staining (Figure 5D) showed that NR6A1 promoted the neural cell‐specific marker MAP2 expression as well as proliferating cell nuclear antigen PCNA expression in mice after feeding of 19 days, whereas the E‐cadherin were suppressed and the staining is very weak, suggesting that NT‐2 cells with NR6A1 transfection may have the capacity to proliferate and transform into neural cells in tumor xenografts. NT‐2 cell exhibits stem cell‐like properties in terms of morphological and biological functions (proliferation, multipotential differentiation) and genomic and epigenetic status, which can be induced to neural‐like cell under RA treatment. Based on the character of NT‐2 cells, to further clarify the role of NR6A1 on tumor neurogenesis in vitro, we treated NT‐2 cells for a longer time (21 days) with RA to establish the neural‐like cell model. The specific culture process is shown in Figure 5E. The results indicated that the morphology of the cells changed significantly at different stages and NT‐2 cell showed a distinct neuron‐like morphology (Figure 5F). Immunofluorescence analysis confirmed the expression and localization of MAP2 protein in these neuron‐like cells (Figure 5G). Using this RA‐induced cell model, we detected the expression alteration of MAP2 under the NR6A1 interference situation. As shown in Figure 5H,I, in the si‐NC group, the level of MAP2 gradually increased in a time‐dependent way. However, after interference with NR6A1 in NT‐2 cells, the expression of MAP2 was not observed at 21 days of RA induction. These results proved that NR6A1 promotes MAP2 expression in vivo and in vitro, suggesting the gain of neural cell‐like characteristics of testicular tumor with high NR6A1 expression.

### 3.6 The correlation of miR‐196a‐5p/NR6A1/E‐cadherin in RA‐induced NT‐2 cells and clinical TGCTs tissue specimens.

**FIGURE 6:**
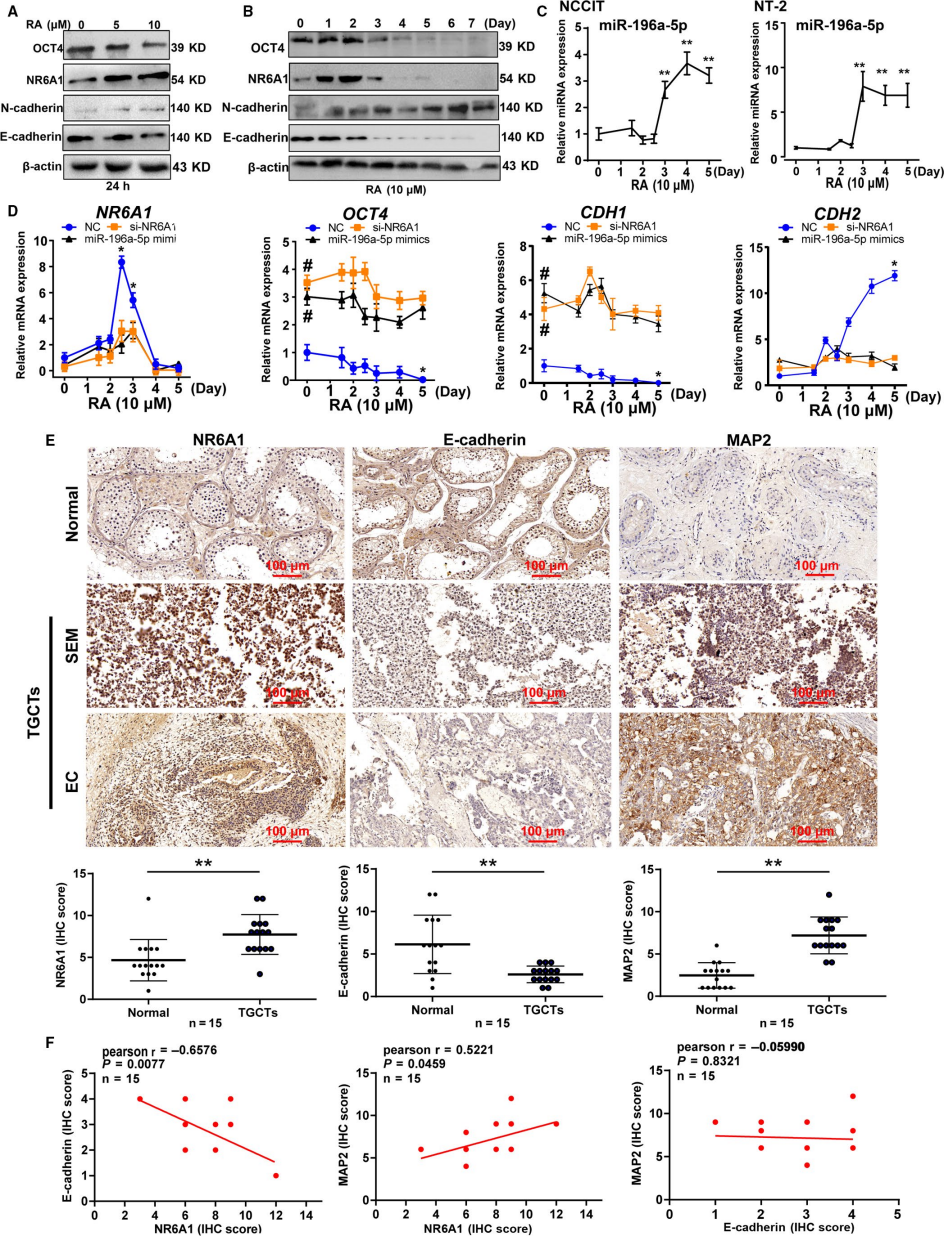
The correlation of miR‐196a‐5p/NR6A1/E‐cadherin in RA‐induced NT‐2 cells and clinical TGCTs tissue specimens. A, Western blot analysis of NR6A1, OCT4, E‐cadherin, and N‐cadherin expression in NT‐2 cells treated with different concentrations of RA for 24 h. B, Western blot analysis of NR6A1, OCT4, E‐cadherin, and N‐cadherin expression in NT‐2 cells treated with 10 μmol/L RA for different numbers of days. C, RT‐qPCR detection of miR‐196a‐5p expression in RA‐treated NCCIT and NT‐2 cells. ** represented *p* < 0.01 compared with cells without RA treatment group. D, The expression alteration of miR‐196a‐5p/NR6A1/E‐cadherin in NT‐2 cells with 10 μmol/L RA treatment for 0‐5 days by RT‐qPCR. * represented *p* < 0.05 compared with RA treated 0 day group; # represented *p* < 0.05 compared with NC mimics group without RA treatment. E, Immunohistochemistry analysis of NR6A1, E‐cadherin, and MAP2 expression in both normal testes and TGCTs. Up panel showed the immunohistochemistry staining of NR6A1, E‐cadherin, and MAP2 protein in both normal testes and TGCTs. Down panel showed the immunohistochemistry scores of NR6A1, E‐cadherin, and MAP2 in both normal testes and TGCTs (n = 15). **p < 0.01. SEM, seminoma. EC, testicular embryonal carcinomas. (F) The correlation between NR6A1 and E‐cadherin (r = −0.6576, *p* = 0.0077), NR6A1 and MAP2 (*r* = 0.5221, *p* = 0.0459), E‐cadherin and MAP2 (*r* = −0.0599, *p* = 0.8321), was analyzed based on immunohistochemistry staining score in clinical TGCTs. The correlation is deemed significant and positive when *p* < 0.05. All data are shown as the means ±SDs of three independent experiments

Since NR6A1 may play a role in neurogenesis of testicular tumors, we next examined the effect of miR‐196a‐5p/NR6A1/E‐cadherin and the correlation of its members in RA induced NT‐2 cells. As shown in Figure 6A,B, the expression of NR6A1 upregulated and peaked at 2 days of RA induction and then abruptly decreased and was barely expressed after 4 days. From 0 to 5 days after RA induction in NT‐2 cell, the E‐cadherin protein levels gradually decreased, and the N‐cadherin protein levels gradually increased. We also observed that the OCT4 protein levels gradually decreased with RA induction, consistent with the literature reports.^32^ At the same time, the expression of miR‐196a‐5p was assessed, and we found that miR‐196a‐5p expression rapidly increased after 2.5 days of RA treatment and remained high until the fifth day both in NT‐2 and NCCIT cells (Figure 6C). These results indicated a reciprocal relationship of miR‐196a‐5p, NR6A1 and E‐cadherin under RA treatment.

In addition, we assessed alterations of NR6A1 in NT‐2 cells with si‐NR6A1 or miR‐196a‐5p mimic transfection under RA induction. As shown in Figure 6D, after miR‐196a‐5p mimic treatment, the expression of NR6A1 no longer increased in cells even under continuous RA induction, consistent with the expression of NR6A1 after si‐NR6A1 treatment. The *CDH1* expression was no longer inhibited, and the promotive effect of RA on *CDH2* (coding gene for N‐cadherin) expression also disappeared, which suggested that NR6A1 or miR‐196a‐5p can regulate the expression level of E/N‐cadherin, and inhibition of NR6A1 may affect the transition from E‐cadherin to N‐cadherin with RA induction. Clinical histopathologically (Figure 6E), compared with the normal testes group, the high expression of MAP2 was also observed in TGCTs with high level of NR6A1 (*p* < 0.01), whereas the E‐cadherin was low in TGCTs (as measured by the IHC score). The Pearson correlation analysis based on IHC score showed that NR6A1 and E‐cadherin had a negative correlation (*r* = −0.6576, *p* = 0.0077), and NR6A1 and MAP2 had a positive correlation (*r* = 0.5221, *p* = 0.0459). The expression correlation between E‐cadherin and MAP2 was not significant (*r* = −0.0599, *p* = 0.8321) (Figure 6F). Moreover, we analyzed the association between the NR6A1/E‐cadherin/MAP2 expression (high or low) and clinicopathological parameters of TGCTs patients, and the results (Table S1) showed that high expression of NR6A1 and MAP2 was positively correlated with lymph node involvement (*p* < 0.05), and high E‐cadherin expression level was negatively correlated with lymph node involvement (*p* < 0.05), tumor size (*p* < 0.05) and metastasis (*p* < 0.05). Besides, high MAP2 expression was positively correlated with pathological type (*p* < 0.05).

## 4 DISCUSSION

Several research has confirmed that tumor cells can produce functional neurons and play an important role in the formation and development of cancer, and nerves are considered to be components of cancer microenvironment.^19^ Cell lines from solid cancers have also observed the characteristics of neural progenitor cells.^17^ These studies suggested tumorigenesis represents a process of gradual loss of cell or lineage identity and gain of characteristics of neural cells. Targeting cancer neurogenesis may be promising for cancer biotherapy. Therefore, the molecular targets with a capacity to promote or inhibit neurogenesis and neural differentiation within tumor cells are of therapeutic importance.

NR6A1 is a member of the nuclear receptor family and also known as transcriptional repressor.^33^ Sequence comparisons indicate that NR6A1 is highly conserved among species.^34^ Studies in mouse embryonic development have shown that NR6A1 is involved in several distinct developmental processes, including possible primordial germ cell differentiation and early development, late development of the gastrula, body axis formation, and neurogenesis.^29^, ^35^, ^36^ A lack of NR6A1 in mouse embryos leads to complete opening of the neural tube and affects the development of the midbrain, which eventually causes embryonic lethality at E10.5.^36^ Recently, Cheng et al reported that NR6A1 is highly expressed in various tumors and might be a new Cancer‐Testis antigen.^37^ However, whether NR6A1 plays a role in tumor neurogenesis and the specific mechanism is unclear. In the present study, NR6A1 was identified as a target of miR‐196a‐5p in TGCTs. We verified the reciprocal correlation between miR‐196a‐5p and NR6A1 on cell proliferation, migration, and invasion in NT‐2 and NCCIT testicular tumor cells, and proved that NR6A1 can directly bind to the *CDH1* gene promoter region by recognizing the DR0 site and simultaneously recruiting the methyltransferase Dnmt1 to inhibit the expression of E‐cadherin and promote the switch of E‐cadherin expression to N‐cadherin expression. It is well known that E‐cadherin plays an important role in establishing cell‐cell junction structures during embryogenesis and is essential for ES cell colony formation.^38^, ^39^ The neuroectodermal cells lose E‐cadherin expression and begin to express N‐cadherin is also a crucial step in the neurodevelopmental process.^40^ Some studies have indicated a negative correlation between E‐cadherin and N‐cadherin expression in neural stem cells.^41^, ^42^ Thus, our works suggested that NR6A1 may play a role in testicular tumor neurogenesis.

We next detected the microtubule‐associated protein 2 (MAP2), a neuronal cytoskeleton regulator, in testicular tumor xenografts, and found that NR6A1 can promote MAP2 expression. MAP2 is a proven marker of human glioma and is used for diagnostic and grading purposes.^43^, ^44^ The data from our animal experiment indicated that the tumor with NR6A1 overexpression gains the neural cell‐like characteristics. Subsequently, using in vitro RA‐induced differentiation of NT‐2 cell model, we demonstrated that when NR6A1 was knocked down, the MAP2 protein did not appear after 21 days of RA induction, implying that NT‐2 cells with NR6A1 silencing could lead to the arrest or delay of NT‐2 differentiation into neuron‐like cells. We also observed the reciprocal expression between miR‐196‐5p and NR6A1, NR6A1 and E‐cadherin under RA induction, and the data confirmed their regulatory relationship in testicular tumor cells. Histopathologically, compared with the normal testes group, the high expression of MAP2 was also observed in clinical tissues of TGCTs with high level of NR6A1, whereas the E‐cadherin was low in TGCTs. And the score of IHC indicated that NR6A1 is negatively correlated with E‐cadherin, and positively correlated with MAP2, respectively. Meanwhile, high expression of NR6A1 and MAP2 was positively correlated with lymph node involvement, and high E‐cadherin expression level was negatively correlated with lymph node involvement, tumor size, and metastasis. These results demonstrated that the neurogenesis of testicular tumor cell requires regulation of miR‐196‐5p/NR6A1/E‐cadherin pathway.

In summary, our findings reveal a novel regulatory pathway of miR‐196a‐5p/NR6A1/E‐cadherin in inhibiting the proliferation, migration, invasion, and neurogenesis of testicular tumor cell in vivo and in vitro, which provides a potential biomarker for targeted TGCTs therapy.

## CONFLICT OF INTEREST

None.

## AUTHOR CONTRIBUTIONS

DL designed the research and wrote the manuscript. XL performed the research and wrote the manuscript. ZF, YL, ZL, ZZ, XY, JW, and ZM performed the research. LY revised the manuscript. All authors were involved in analyzing the data. All authors read and approved the manuscript.

## Supporting information

Supplementary Material

## Data Availability

The data that support the findings of this study are available from the corresponding author upon reasonable request.
