# Supplementary material for: microRNA‐196a‐5p inhibits testicular germ cell tumor progression via NR6A1/E‐cadherin axis
Source: Cancer Med. 2020 Oct 9;9(23):9107–22. doi: 10.1002/cam4.3498 (PMC7724306; doi:10.1002/cam4.3498)
Supplement: Supplementary file 1 — Supplementary Material [file CAM4-9-9107-s001.docx]

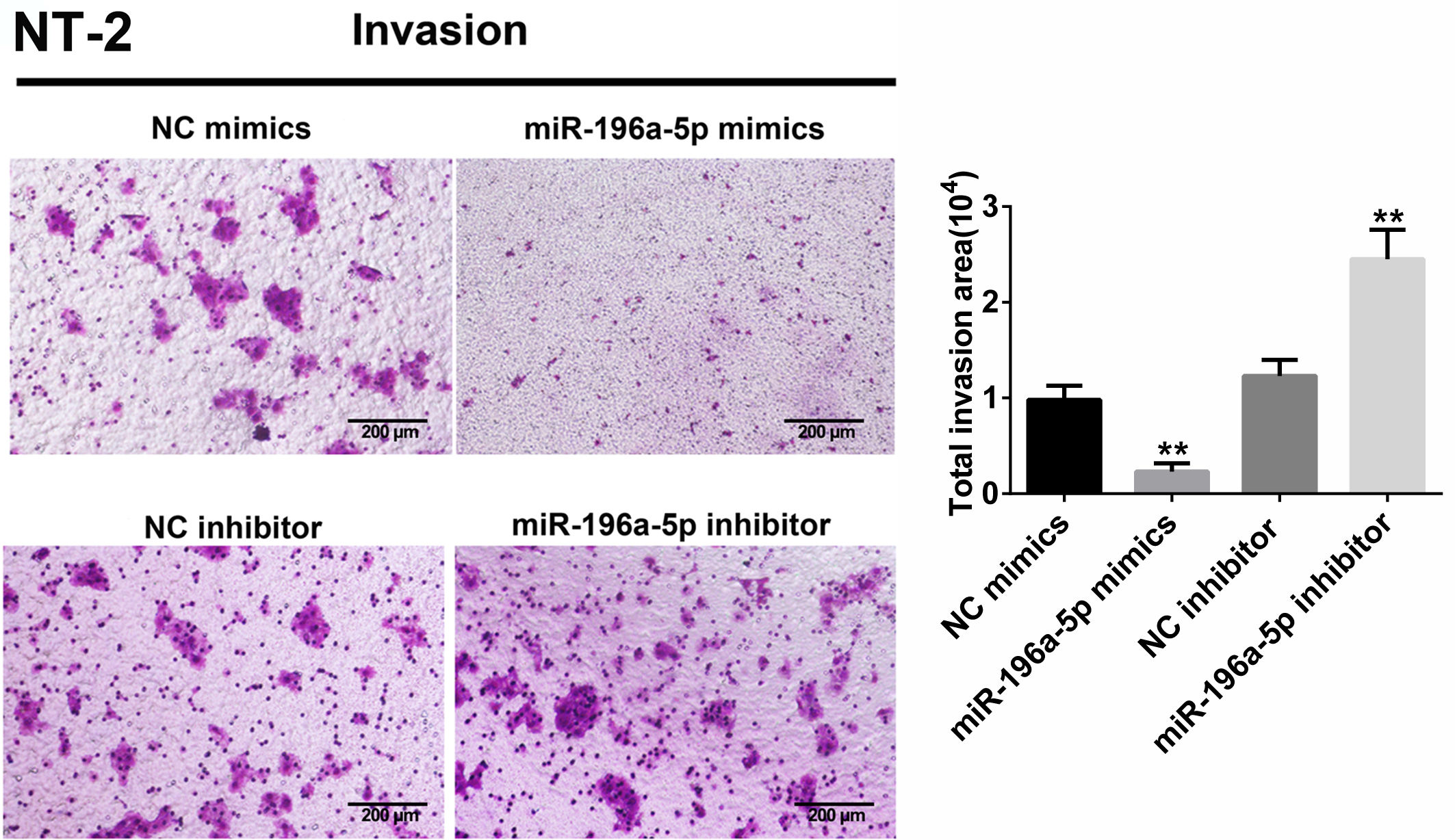


**Figure S1. The inhibition of miR-196a-5p on invasion of NT-2 cells by transwell assay analysis.** ** represented compared with NC mimics or inhibitor group, *p*<0.01


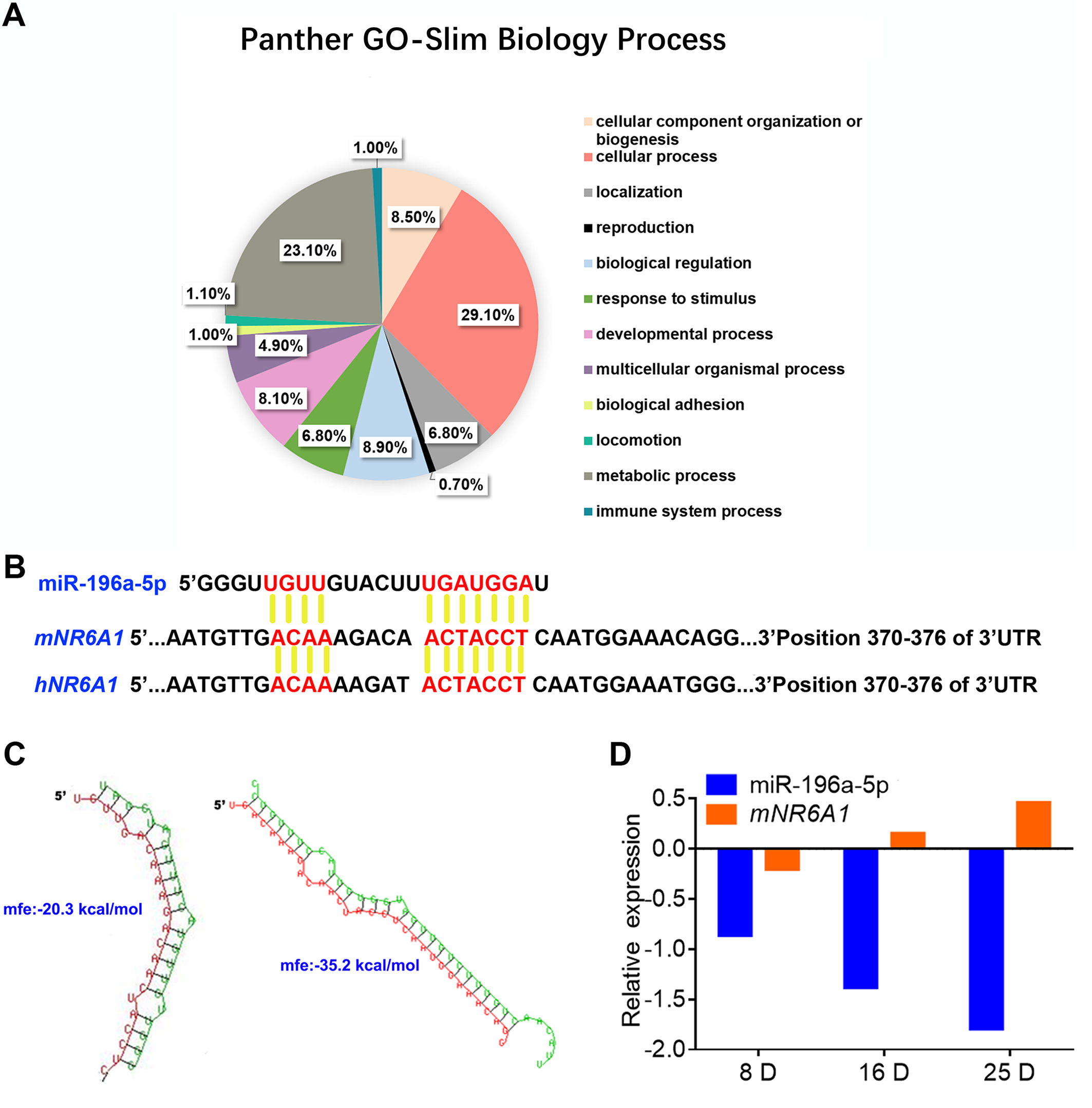


**Figure S2. miR-196a-5p targeted NR6A1 for inhibition.** (A) [Biological](file:///D:\youdaoDict\7.5.2.0\resultui\dict\?keyword=biological)[process](file:///D:\youdaoDict\7.5.2.0\resultui\dict\?keyword=process) analysis for all predicted target genes (364) of miR-196a-5p by GO database analysis. (B) The predicted miR-196a-5p binding sites in 3'UTR of NR6A1 mRNA sequence by TargetScan. (C)Analysis of the minimum hybridization free energy. Left: the secondary structure of single‑stranded miR-196a-5p mRNAs generated by RNAFold WebServer; Right: the minimum hybridization free energy of miR-196a-5p and NR6A1 mRNA by RNAhybrid analysis. (D)The reciprocal expression of miR-196a-5p and NR6A1 on different developmental days in mice by NCBI GEO dataset analysis.


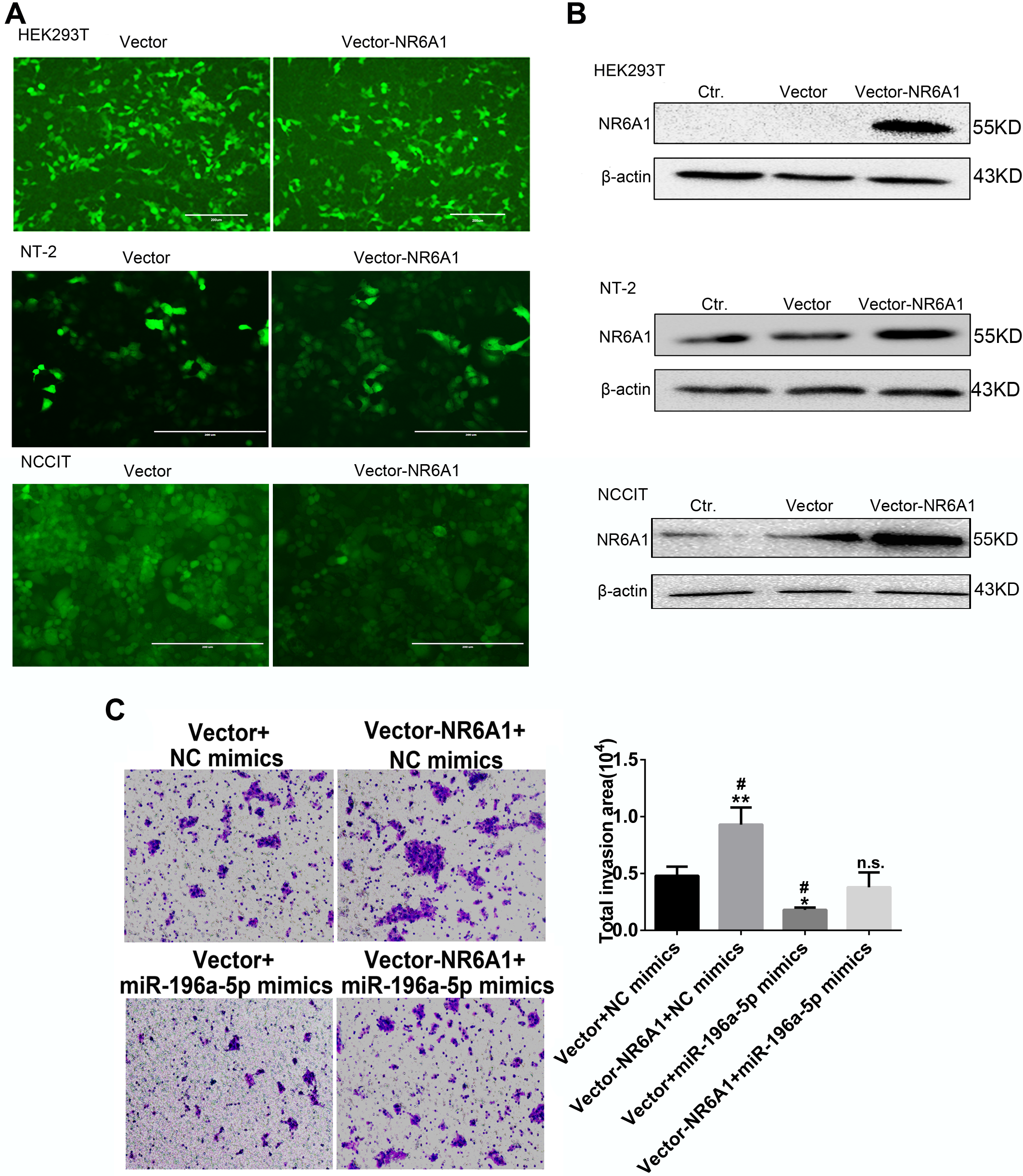


**Figure S3. Cooverexpression of NR6A1 and miR-196a-5p mimics rescued the invasion ability of NT-2 cells.** (A)Transfection efficiency analysis of pLv[Exp]-EGFP: T2A: Puro-EF1A>NR6A1 in HEK293T, NT-2 and NCCIT cells by fluorescence microscopy. (B)Western blot analysis of NR6A1 in infected cells. Vector indicates virus packaged using pLv[Exp]-EGFP: T2A: Puro-EF1A>; Vector-NR6A1 indicates virus packaged with pLv[Exp]-EGFP:T2A:Puro-EF1A>NR6A1. (C) Cooverexpression of NR6A1 and miR-196a-5p mimics rescued the invasion ability of NT-2 cells, as evidenced by the transwell assay. * represented *p*<0.05, ** represented *p*<0.01 compared with Vector + NC mimics group, and # represented *p*<0.05 compared with Vector-NR6A1 + miR-196a-5p mimics group; n.s. represented no significant changes compared with Vector + NC mimics group. All data are shown as the means ± SDs of three independent experiments.


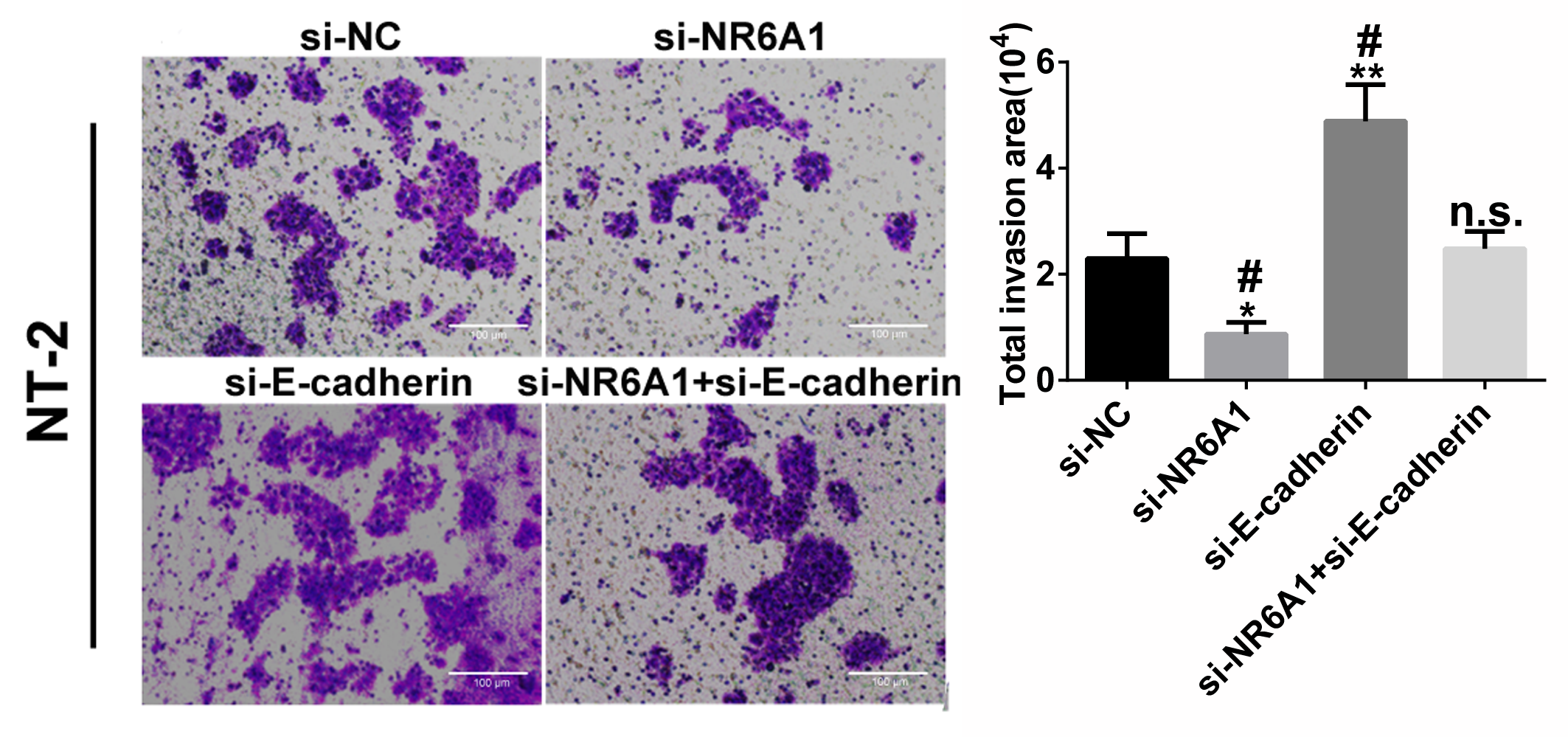


**Figure S4. The effect of co-interference with NR6A1 and E-cadherin on the invasion ability of NT-2 cells by transwell assay analysis.** * represented *p*<0.05, ** represented *p*<0.01 compared with si-NC group, and # represented *p*<0.05 compared with si-NR6A1 + si-E-cadherin group; n.s. represented no significant changes compared with si-NC group. All data are shown as the means ± SDs of three independent experiments.

**Supplementary Table 1 Clinical correlation of NR6A1/E-cadherin/MAP2 expression in the 15 patients with TGCTs**

| Clinicopathological parameters | Case  （n） | NR6A1 | | | E-cadherin | | | MAP2 | | |
| --- | --- | --- | --- | --- | --- | --- | --- | --- | --- | --- |
|  |  | Low | High | *P*-values | Low | High | *P*-values | Low | High | *P*-values |
| Seminoma | 6 | 3 | 3 | 0.2352 | 2 | 4 | 0.0889 | 3 | 3 | 0.0440* |
| Non-seminoma | 9 | 1 | 8 |  | 7 | 2 |  | 0 | 9 |  |
| Age | | | | | | | | | | |
| ≤45 | 9 | 4 | 5 | 0.1033 | 5 | 4 | 0.5804 | 2 | 7 | 1.0000 |
| > 45 | 6 | 0 | 6 |  | 5 | 1 |  | 1 | 5 |  |
| Tumor size (cm) | | | | | | | | | | |
| ≤1 | 9 | 3 | 6 | 0.2286 | 3 | 6 | 0.0278* | 3 | 6 | 0.2286 |
| > 1 | 6 | 0 | 6 |  | 6 | 0 |  | 0 | 6 |  |
| Lymph node | | | | | | | | | | |
| - | 6 | 4 | 2 | 0.0110* | 1 | 5 | 0.0110* | 3 | 3 | 0.0440* |
| + | 9 | 0 | 9 |  | 8 | 1 |  | 0 | 9 |  |
| Metastasis(NSE) | | | | | | | | | | |
| - | 11 | 3 | 8 | 0.5165 | 9 | 2 | 0.0110* | 3 | 8 | 0.5165 |
| + | 4 | 0 | 4 |  | 0 | 4 |  | 0 | 4 |  |

N: number of cases in each group; *, *P* < 0.05.
